# Impact of anxiety and fear for COVID-19 toward infection control practices among Thai healthcare workers

**DOI:** 10.1017/ice.2020.280

**Published:** 2020-06-08

**Authors:** Anucha Apisarnthanarak, Piyaporn Apisarnthanarak, Chanida Siripraparat, Pavarat Saengaram, Narakorn Leeprechanon, David J. Weber

**Affiliations:** 1 Division of Infectious Diseases, Faculty of Medicine, Thammasat University, Khlong Nueng, Prathum Thani, Thailand; 2 Division of Diagnostic Radiology, Department of Radiology, Faculty of Medicine Siriraj Hospital, Mahidol University, Bangkok, Thailand; 3 Manorom Hospital, Bangkok, Thailand; 4 Bumrungrad Hospital, Bangkok, Thailand; 5 Department of Opthalmology, Rutnin Hospital, Bangkok, Thailand; 6 Division of Infectious Diseases, University of North Carolina, Chapel Hill, North Carolina, United States

The emergence of COVID-19 is the most challenging threat to international public health.^1,2^ The epidemic had a vast impact on healthcare personnel (HCP), who are at risk for contracting diseases and transmission to their patients and families. The uncertainty about the mode of transmission, including infectivity of asymptomatic and presymptomatic patients, may have created substantial stress in HCP who provide care for known or suspected COVID-19 patients.^3^ This anxiety may lead to substandard care for patients that may negatively impact patient safety. As of April 26, 2020,^4^ there were 2,951 patients with COVID-19 in Thailand, and 99 HCP had contracted COVID-19 during patient care. Few data are available concerning the impact of HCP emotions (eg, anxiety and fear) toward infection prevention practices.^5,6^ To evaluate the HCP emotions for COVID-19 toward infection prevention practices at 4 hospitals, a we conducted a survey.

This survey was performed at 2 university hospitals (Thammasat University Hospital, Khlong Nueng, Pratum Thani, Thailand, and Siriraj Hospital, Bangkok, Thailand) and 2 private hospitals (Bumrungrad Hospital, Bangkok, and Rutnin Hospital, Bangkok) from March 1 through 31, 2020. HCP in the general medicine, ophthalmology, and radiology departments were invited to participate using a standardized data collection tool. Data collected included HCP demographics, perception of risks to contract COVID-19, confidence in policies and hospital preparedness plans for COVID-19 (eg, policy or adequacy of personal protective equipment [PPE]), confidence in knowledge of disease transmission and infection prevention practices, sources for COVID-19 news, HCP emotions (eg, anxiety and fear), their infection prevention practices including hand hygiene, wearing a mask and PPE, physical distancing, willingness to see admitted patients and willingness to accept new patients, as well as suggestions on how to deal with emotions. Respondents rated the frequency of perception of risk, confidence on knowledge, and hospital preparedness plan on a scale of 1 to 5, where 1 indicates “no risk/no confidence” and 5 indicates “very risky/very confidence.” They rated infection prevention practices on a scale of 1 to 5, where 1 indicates “never use” and 5 indicates “always use.” Confidence and regular use were assessed using a rating of 4 (confident and almost always) or 5 (very confident and always). We used the Generalized Anxiety Disorder 7-item (GAD-7) scale to categorized HCP anxiety; it is a self-rated fear scale from 1 to 10, where 1 indicates “no fear” and 10 indicates “extremely fearful.” Categorization of GAD-7 score followed the original scale (eg, 0–4 = minimal anxiety; 5–9 = mild anxiety; 10–14 = moderate anxiety; and >14 = severe anxiety),^7^ and a self-reported fear score >6 was categorized as fear of COVID-19.

All analyses were performed using SPSS, version 19 software (IBM, Armonk, NY). The χ^2^ or Fisher exact test was used to compare categorical variables. Mann-Whitney U tests were used for all continuous data. All *P* values were 2-tailed, and *P* < .05 was considered statistically significant. A multivariate analysis was used to evaluate factors associated with emotions and the impact of emotions on infection prevention practices. Variables that had a significant level of *P* < .20 and variables with prior significance in univariate analysis were entered into multivariate logistic regression models. Adjusted odd ratios (aORs) and 95% confidence intervals (CIs) were calculated.

In total, 160 HCP participated in this survey (n = 40 HCP per hospital). Among them, 95 HCP (59%) were women, and the median age was 32 years (range, 23–62 years). Most HCP respondents were physicians (52 of 160, 32%), nurses (45 of 160, 28%), or nurse assistants (16 of 160; 1%). Most HCP categorized themselves as being at high risk of contracting COVID-19 (144 of 160, 90%) and for being quarantined (136 of 160, 85.5%). Most were fearful of COVID-19 (144 of 160, 90%), and 68 HCP (42.5%) were categorized as having at least a mild anxiety disorder (Table 1). On the other hand, fewer HCP reported confidence in hospital infection prevention policy (125 of 160, 78%), adequacy of PPE (119 of 160, 74.4%) and confidence in their knowledge of disease transmission (118 of 150, 74%), and infection prevention (121 of 160, 75.6%). Hand washing (152 of 160, 95.6%), wearing a mask and PPE (148 of 160, 93.1%), physical distancing at hospital (128 of 160, 82%) were reported at high rates, while willingness to see admitted patients (78 of 160, 48.7%) and willingness to accept new patients (73 of 160, 45.1%) were less likely. The sources of COVID-19 news were social media such as the Line application or Facebook (155 of 160, 97%), hospital news (115 of 160, 71.8%), and television (105, 90%). HCP had low confidence in social media COVID-19 news (105 of 160, 65.6%), but almost all HCP had very high confidence in non–social media COVID-19 news (155 of 160, 96.8%).

**Table 1.** Healthcare Personnel Characteristics, Emotions, and Infection Prevention Practices

Variable	No. (%) (N = 160)
Age, median y (range)	32 (23–62)
Sex, female	95 (59)
**Occupation**	
Physicians	52 (32)
Nurse	45 (28)
Nurse assistant	16 (1)
Others^a^	**47 (29)**
Direct contact with COVID-19 patients	82 (51.6)
Perceived high risk of contracting COVID-19 patients	144 (90)
Perceived high risk of being quarantine	136 (85.5)
Confidence in hospital preparedness policy for COVID-19	125 (78)
Confidence in hospital stockpile policy for PPE	119 (74.4)
Confidence in knowledge of COVID-19 diseases transmission	118 (73.8)
Confidence in knowledge of COVID-19 infection preventions	121 (75.6)
**Sources of COVID-19 news**	
Social media	144 (90)
Line application	135 (84.3)
Facebook	116 (72.1)
Instragram	115 (71.8)
Hospital news	105 (90)
Television news	144 (90)
**Fearful for COVID-19**	
**GAD-7 Score**	
**Minimal anxiety**	**51 (31.8)**
Mild anxiety	37 (23.1)
Moderate anxiety	23 (14.4)
Severe anxiety	8 (5)
**Infection prevention practices**	
Hand washing	152 (95.6)
Wearing mask and PPE	148 (93.1)
Willing to see admitted patients during epidemics	78 (48.7)
Willing to accept new patients during epidemics	73 (45.1)
Social distancing in hospital	128 (82)
Social distancing in community	125 (78)
**Suggestions to improve HCP emotions**	
Hospital policy on adequate PPE stockpile	114 (71)
Ongoing education on diseases transmission and preventions	125 (77)
Mindfulness practices	89 (55)
Workshop to share knowledge and experience	75 (28)
Increase incentive for being at risk	35 (21.8)

Note. PPE, personal protective equipment; HCP, healthcare personnel; GAD-7, Generalized Anxiety Disorder-7.

a Technician, nursing aids, pharmacist, clinical pharmacist, physical therapists.

By multivariate analysis, no factor was associated with anxiety and fear. However, HCPs who reported fear and anxiety were more likely to wash hands (aOR, 12.4; 95% CI,1.5–69.9) and to wear a mask and PPE (aOR, 7.8; 95% CI, 1.2–45.9), but they were less likely to be willing to see admitted patients (aOR, 0.45; 95% CI, 0.14–0.89) and to accept new admissions during epidemics (aOR, 0.65; 95% CI, 0.24–0.96). Suggestions to improve anxiety and fear including improvement of the hospital policy on PPE (114 of 160, 71%), ongoing reliable infection prevention education during epidemics (124 of 160, 77%), and mindfulness practices (89 of 160, 55%). Notably, all HCP categorized as having mild-to-severe anxiety reported fear of COVID-19.

In this study, most HCP were overwhelmed with fear and anxiety during the COVID-19 pandemic. Although these emotions lead to more appropriate infection prevention practices, most HCP were not willing to accept new patients or to see admitted patients during epidemics, which may compromise patient safety. Despite the limitations of self-reported surveys and the sample size in this study, our findings support the need for hospitals to have good preparedness policies, particularly regarding the PPE stockpile, and ongoing education on disease transmission and infection prevention. Additional studies to evaluate strategies to improve psychological support among HCP during epidemics will help balance HCP safety without compromising the standard of patient care during epidemics.
